# Role of neuromedin B and its receptor in the innate immune responses against influenza A virus infection in vitro and in vivo

**DOI:** 10.1186/s13567-019-0695-2

**Published:** 2019-10-10

**Authors:** Guihong Yang, Huipeng Huang, Mengyao Tang, Zifeng Cai, Cuiqin Huang, Baomin Qi, Ji-Long Chen

**Affiliations:** Key laboratory of Fujian-Taiwan Animal Pathogen Biology, College of Animal Sciences, Fujian Agricultural and Forestry University, Fujian, 350002 China

## Abstract

The peptide neuromedin B (NMB) and its receptor (NMBR) represent a system (NMB/NMBR) of neuromodulation. Here, it was demonstrated that the expression of NMBR in cells or murine lung tissues was clearly upregulated in response to H1N1/PR8 influenza A virus infection. Furthermore, the in vitro and in vivo activities of NMB/NMBR during PR8 infection were investigated. It was observed that A549 cells lacking endogenous NMBR were more susceptible to virus infection than control cells, as evidenced by the increased virus production in the cells. Interestingly, a significant decrease in IFN-α and increased IL-6 expression were observed in these cells. The role of this system in innate immunity against PR8 infection was probed by treating mice with NMB. The NMB-treated mice were less susceptible to virus challenge, as evidenced by increased survival, increased body weight, and decreased viral NP expression compared with the control animals. Additionally, the results showed that exogenous NMB not only enhanced IFN-α expression but also appeared to inhibit the expression of NP and IL-6 in PR8-infected cells and animals. As expected, opposing effects were observed in the NMBR antagonist-treated cells and mice, which further confirmed the effects of NMB. Together, these data suggest that NMB/NMBR may be an important component of the host defence against influenza A virus infection. Thus, these proteins may serve as promising candidates for the development of novel antiviral drugs.

## Introduction

Influenza A viruses (IAVs) invade the respiratory tract, causing direct damage via viral replication and indirect damage via the host’s excessive defensive, production of inflammatory cytokines, called the cytokine storm [1]. Cytokine dysregulation contributes to the pathogenesis of H1N1, H5N1 and H7N9 viruses [2, 3] by inducing an imbalance in the host regulatory network, which results in severe complications and ultimately high mortality rates [4, 5]. The most important methods for preventing and controlling IAV are antiviral treatments and annual vaccination. However, IAV antigens can mutate rapidly through the processes of antigenic drift and antigenic shift. As a result, drug-resistant viruses are continually emerging [6]. Over time, drug-resistant subtypes of IAV have been observed to escape the actions of antiviral drugs [7, 8]. Several drugs, such as amantadine and rimantadine, have been withdrawn from the market as a result of their reduced efficacy [9–12]. Currently, available antiviral drugs have several disadvantages: many negative side effects [13, 14], increased drug resistance, and single-target modes of action [7, 9, 10, 13, 15]. Thus, anti-influenza drug development has increasingly focussed on host immunoregulation [16]. In response to viral infection, rapid induction of cytokines, including type I interferon (IFN-α/β), is central to the establishment of innate antiviral immunity [17, 18]. It has been demonstrated that neuroendocrine molecules are capable of regulating cytokine responses through auto- or self-secretions [19]. Therefore, neuroendocrine molecules may serve as promising candidates for the development of novel and potent antiviral drugs. Such members include neuromedin B (NMB), neuromedin U, and neuropeptide S. In particular, NMB has been shown to directly regulate IL-6 expression in tissues as well as its secretion into the serum of animals [20, 21]. Therefore, the potential antiviral efficacy of NMB warrants further investigation.

To date, few reports have evaluated the role of NMB and NMBR in the process of IAV infection in animals. To this end, the potential anti-influenza activity of NMB and its receptor NMBR were examined here. Since it is very difficult to obtain natural NMB, the NMB used here was chemically synthesized. Additionally, a synthetic NMBR antagonist (NMBRA) was employed. First, NMB and NMBR expression was measured in PR8-infected cells and animals. Next, cells with a stable knockdown of NMBR were generated to assess its role in the host responses to IAV infection. NMBR-knockdown cells were more susceptible to PR8 infection than control cells. In addition, the effects of NMB and NMBRA treatment on PR8 infection both in vitro and in vivo were determined, which provided initial evidence that NMB and NMBR exert anti-H1N1 IAV effects.

## Materials and methods

### Animals and ethics statement

Specific pathogen-free (SPF) C57BL/6J mice at an age range of 5–6 weeks used in the present study were obtained from the WUSHI animal centre (Shanghai, China). The animals were housed in sterile cages under laminar airflow hoods in a SPF room with a 12 h:12 h light–dark schedule. Animals were provided autoclaved feed and water ad libitum. This study was carried out in strict accordance with the Regulations for the Administration of Affairs Concerning Experimental Animals approved by the State Council of China. The study protocol was approved by the Research Ethics Committee of the College of Animal Science, Fujian Agriculture and Forestry University, under Permit Number PZCASFAFU2014002. All surgeries were performed under ether anaesthesia to minimize suffering and discomfort as much as possible. All animals received humane care in compliance with the university’s guidelines.

### Virus, cell lines and peptides

Influenza virus strain A/Puerto Rico/8/1934 (H1N1) (hereafter referred to as PR8) was propagated in SPF chicken embryos as previously described [22]. Viruses were then harvested and preserved at −80 °C prior to use. Virus titres were determined using a standard plaque-forming unit (PFU) assay in MDCK cells. The titre of the virus stock obtained was 6.5 × 10^6^ PFU/mL.

The 293T, A549 and MDCK cells used in the present study were purchased from American Type Culture Collection. Cells were cultured at 37 °C with 5% CO_2_ in Dulbecco’s modified Eagle’s medium (DMEM) supplemented with 10% (v/v) foetal bovine serum (FBS) (Gibco, US), 100 units of penicillin G, and 100 µg of streptomycin.

The NMB peptide and NMBR antagonist (NMBRA) were synthesized by manual solid-phase synthesis using standard Fmoc chemistry as previously described [23]. Synthetic NMB and NMBRA were dissolved in a 0.9% NaCl solution at doses of 1 nM and stored at −20 °C until use. The doses used here were selected based on a previous report [24] in addition to our own previous experiments.

### shRNA-based knockdown of NMBR and generation of cell lines

Short hairpin RNA (shRNA)-based knockdown cell lines were generated by transfection of A549 cells with lentiviral vectors expressing specific shRNAs in a previously described pSIH-H1-GFP vector [22]. Sequences for NMBR shRNA were designed using Invivogen’s online shRNA wizard. The shRNA targeting the NMBR gene (NMBR-shRNA), 5′-GGCAATTGCATGATTGACTCA-3′, was designed. A control shRNA targeting the luciferase gene (NC-shRNA), 5′-CTTACGCTGAGTACTTCGA-3′, was used. The oligonucleotide duplex for each target was cloned into the pSIH-H1-GFP shRNA expression vector. Plasmids expressing NC- and NMBR-shRNA were cloned. Stable A549 cells expressing NMBR-shRNA (sh-NMBR cells) or NC-shRNA (sh-Luciferase cells) were generated using viral spin infection as described previously [25]. The above vector expresses GFP under the control of the CMV promoter to allow monitoring of transfection efficiency. To measure the knockdown efficiency, RT-PCR, quantitative RT-PCR (qRT-PCR) and Western blotting were performed.

### Isolation of murine bone marrow-derived macrophages (BMDMs)

Primary BMDMs were isolated as previously described [26]. Both femurs were dissected, and bone marrow was flushed out from the medullary cavities with Dulbecco’s phosphate-buffered saline containing 1 × penicillin/streptomycin. Cells were centrifuged for 10 min at 1200 rpm at room temperature and then resuspended in complete medium (DMEM supplemented with 10% FBS (v/v), 1% penicillin/streptomycin, and 50 ng/mL M-CSF (recombinant murine M-CSF, RP-8615, Invitrogen Inc., USA)). The cells were cultured at 37 °C under 5% CO_2_ for 5 days with complete medium that was changed twice weekly. Approximately 5 × 10^6^ BMDMs were obtained from each mouse. The viability of BMDMs was > 95%, as confirmed by trypan blue exclusion staining.

### Virus infection and treatment in vitro

In vitro infection of cells with the PR8 virus was performed under biosafety level 2 (BSL-2) laboratory conditions. 293T, A549, sh-NMBR, sh-Luciferase and BMDM cells were seeded in 6-well plates at 37 °C in 5% CO_2_. When the cells grew to a density of approximately 80–90% confluence, the cells were infected with PR8 at the indicated multiplicity of infection (MOI) with gentle agitation every 15 min. After adsorption at 37 °C for 1 h, the cells were washed with phosphate-buffered saline (PBS) and cultured in DMEM containing 2 μg/mL trypsin (Sigma-Aldrich). 293T and A549 cells were harvested at 0, 3, 6, 9, and 12 h post-infection (hpi) for NMB and NMBR expression analysis by RT-PCR and qRT-PCR. The sh-NMBR cells and sh-Luciferase cells were harvested at either 0, 6, or 12 hpi for gene expression analysis by RT-PCR and qRT-PCR and protein expression by WB. Supernatants of sh-NMBR cells and sh-Luciferase cells were harvested at 12, 24, 36, 48 and 64 hpi for virus titration. The viral titres were determined by plaque assay.

One hour after infection with PR8, the BMDMs and A549 cells were incubated with either 1 nM NMB or NMBR antagonist (NMBRA) and cultured at 37 °C in 5% CO_2_. Meanwhile, the mock-treated cells were incubated with DMEM without NMB or NMBRA. Cells were harvested at 16 hpi for gene expression analysis by RT-PCR and qRT-PCR.

### Infection of mice and treatments

Six groups of mice were set up: group 1 (PR8-Mock), group 2 (PR8-NMB +), group 3 (PR8-NMBRA+), group 4 (PR8 + Mock), group 5 (PR8 + NMB +), and group 6 (PR8 + NMBRA+). Each group contained twenty-five mice, with each mouse having a mass of approximately 20–22 g. For this experimental setting, the animals in groups 1, 2, and 3 were mock-treated with 100 μL SPF chick embryo allantoic fluid, and the animals in groups 4, 5, and 6 were anaesthetized and inoculated intranasally with 6.5 × 10^4^ PFU of PR8 virus. On the day after infection, the animals in groups 1 and 4 were mock-treated with 100 μL 0.9% NaCl solution. Simultaneously, the animals in groups 2 and 5 were injected with 1 nM NMB in a volume of 100 μL, and the animals in groups 3 and 6 received 100 μL 1 nM NMBRA. All mice were monitored daily for signs of flaccid paralysis, and any deaths were recorded. Ten mice from each group were euthanized on 3 dpi, from which lung samples were collected and immediately flash-frozen in liquid nitrogen and then stored at −80 °C until future RNA extraction. All other animals in each group were monitored daily for up to 10 days.

### RT-PCR and qRT-PCR

Total RNA was extracted from PR8-infected and control cells at the indicated time points. In addition, RNA was extracted from mouse lung tissues at 3 dpi using Trizol (TIANGEN Biotech, China) according to the manufacturer’s instructions. Equal amounts of RNA (2 µg) were reverse transcribed into cDNA utilizing M-MLV Reverse Transcriptase (Promega, USA). The cDNA was analysed by qRT-PCR using the TransStart Green qPCR SuperMix (TransGen Biotech, China) and RT-PCR using rTaq DNA polymerase (Takara Bio, Japan). Human β-actin and glyceraldehyde-3-phosphate dehydrogenase (GAPDH) were used as references for internal standardization. Amplicons from the PCRs were separated on a 1.5% agarose gel, which was stained with nucleic acid stain I (Roche Diagnostics, Mannheim, Germany), photographed, and analysed using the Gene Tools Analysis Software (Syngene, Cambridge, UK). The qRT-PCR data are presented as normalized ratios, which were calculated using the ΔΔCT method with the LightCycler system and software (Roche, Switzerland). All primers used for RT-PCR and qRT-PCR are presented in Tables 1 and 2.

**Table 1:** **Primers used for RT-PCR and qRT-PCR in mouse tissue**

Primer names	GenBank accession no.	Sequence (5′–3′)
NMB sense	NM_001291280.1	CGGTCACTTCATGGGCAAG
NMB antisense		GAGCTTTCTTTCGCAGGAGGA
NMBR sense	NM_008703.3	CATGCGGAATGTCCCTAACATC
NMBR antisense		CCAAGCTACCAATGCGTGCTAC
β-actin sense	NM_007393.5	AATGGGTCAGAAGGACTCCT
β-actin antisense		ACGGTTGGCCTTAGGGTTCAG
IL-6 sense	NM_001314054.1	TTGCCTTCTTGGGACTGATG
IL-6 antisense		TCTGGCTTTGTCTTTCTTGT
IFN-α sense	NM_206871.2	TCCTGCCTGAAGGACAGGAAGG
IFN-α antisense		AGGGCTCTCCAGACTTCTGCTCTG
NP sense	CY034135.1	TCAAACGTGGGATCAATG
NP antisense		GTGCAGACCGTGCTAAAA

The primers designed for the NP gene listed in this table could also be used in human tissues after virus infection.

**Table 2:** **Primers used for RT-PCR and qRT-PCR in human cells**

Primer names	GenBank accession no.	Sequence (5′–3′)
NMB sense	NM_021077.4	TAAAGAAGGCTCTGGGCGTG
NMB antisense		GGTGACCCAGCCAGAAATCA
NMBR sense	NM_002511.3	ACCTAAATCGTGGGCGTTCA
NMBR antisense		GGCAGGAAATCCCTTTCCCA
GAPDH sense	NM_002046.7	TGGGTGTGAACCATGAGAAGT
GAPDH antisense		AAGGCCATGCCAGTGAGCTT
IL-6 sense	NM_000600.5	ACAAATTCGGTACATCCTCGAC
IL-6 antisense		TGGCTTGTTCCTCACTACTCT
IFN-α sense	J00210.1	TGATCTGCCTCAAACCCACA
IFN-α antisense		ATCTGCTGGATCATCTCATGG

### Western blotting

Lung tissue lysates from the mice were prepared from tissues collected at 3 dpi. Lysates of sh-NMBR cells, sh-Luciferase cells, PR8-infected sh-NMBR cells, and non-infected controls were prepared from samples harvested at 0, 6, and 12 hpi. All lysates were resolved by SDS-PAGE in 10% polyacrylamide gels. Bands were detected using rabbit anti-H1N1-NP (generated in our laboratory) as previously described [27] and anti-hNMBR (ab134141, Abcam, USA). β-Actin was detected using a rabbit anti-actin polyclonal antibody (R019, TransGen Biotech, China). The secondary antibodies for detection were goat anti-mouse (125229, Jackson ImmunoResearch Laboratories, USA) and goat anti-rabbit antibodies (131879, Jackson ImmunoResearch Laboratories). The blots were developed using the FluorChem M Imaging System (ProteinSimple, USA).

### Plaque assay

The supernatant was diluted with medium and added to MDCK cell monolayers in 6-well plates. After 1 h of incubation in 5% CO_2_ at 37 °C, the supernatant was removed, and the covering medium containing 1.5% low-melting-point agarose (Promega, Madison, WI, USA) was then overlaid on the cells. After coagulation of agarose, the maintenance medium was added, and the plates were further incubated for 3 days for plaque formation. Next, the cells were fixed with 10% formalin overnight and stained with crystal violet (1% W/V) for 30 min. The number of plaques was counted.

### Statistical analysis

Survival curves were analysed using the log-rank test (GraphPad Prism5). Other data are presented as mean ± SEM. Differences were considered significant when *P *≤ 0.05. Statistical analysis was completed by one-way ANOVA using SPSS 16.0 (Chicago, IL, USA).

## Results

### Expression of NMB and NMBR is upregulated in 293T and A549 cells following PR8 infection

**Figure 1:**
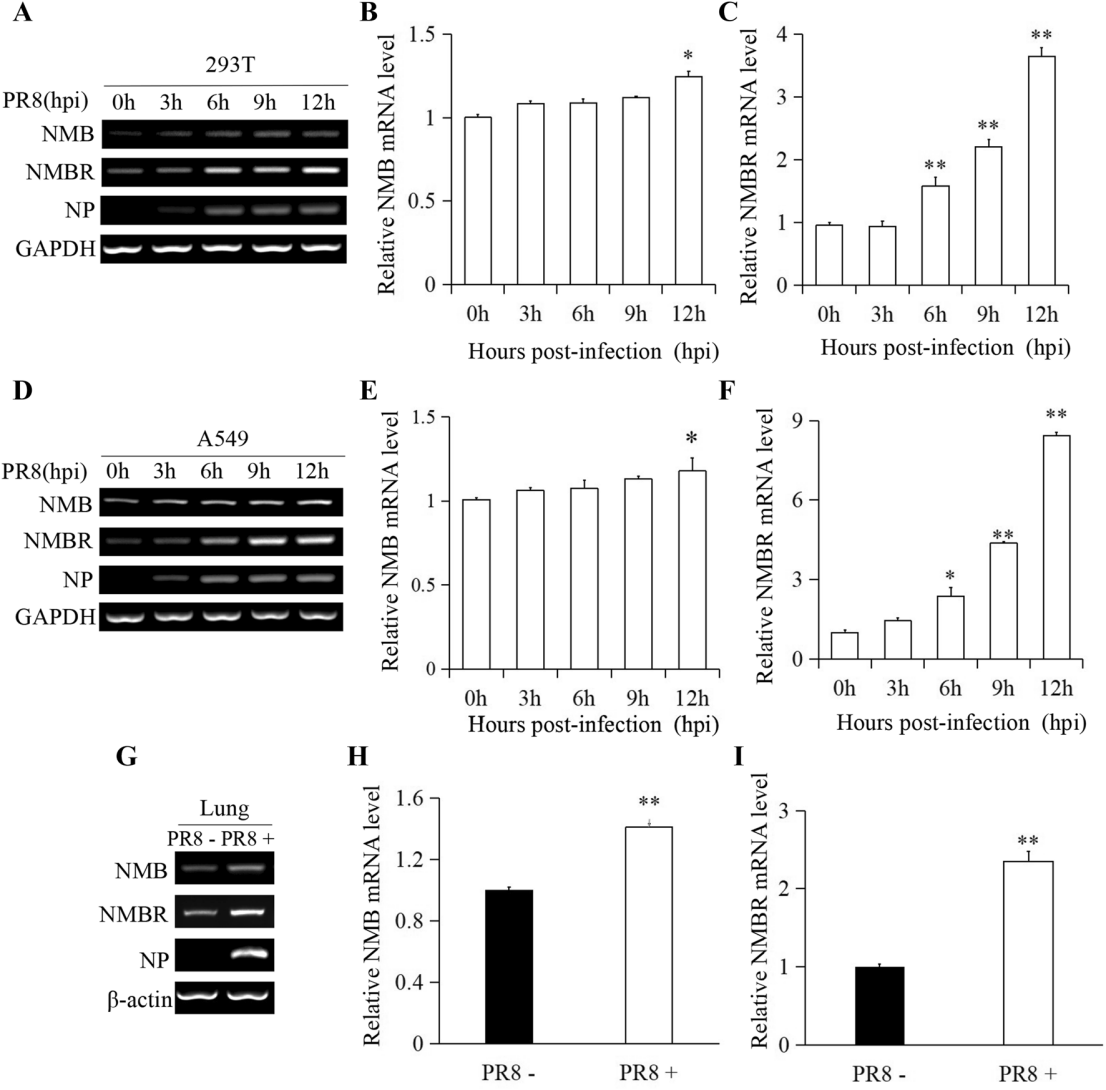
**PR8 induction of NMB and NMBR expression.** 293T and A549 cells were infected with PR8 (MOI = 1) and harvested at 0, 6, and 12 hpi, and lung tissues were harvested at 3 dpi, to measure the expression of NMB and NMBR by RT-PCR and qRT-PCR. **A**–**C** Expression of NMB and NMBR in 293T cells. **D**–**F** Expression of NMB and NMBR in A549 cells. **G**–**I** NMB and NMBR expression in mouse lungs. GAPDH, glyceraldehyde-3-phosphate dehydrogenase; NP, virus nucleoprotein; β-actin or GAPDH were used as reference housekeeping genes for internal standardization. *P < 0.05, **P < 0.01.

To determine the role of NMB and its receptor, NMBR, in the host antiviral response to IAV infection, the expression of NMB and NMBR was analysed in 293T and A549 cells at 0, 3, 6, 9, and 12 hpi. The expression levels of both NMB and NMBR were very low in the mock-treated controls (0 hpi). The expression level of NMB was slightly increased, whereas the expression of NMBR was clearly increased, in 293T cells in response to virus infection (Figure 1A). Consistent with that, the results from qRT-PCR showed that the expression of NMB mRNA was significantly increased at 12 hpi (Figure 1B), and expression of NMBR mRNA was significantly increased at 6, 9, and 12 hpi (Figure 1C). Similar profiles of both NMB and NMBR expression following infection with PR8 were also observed in A549 cells (Figures 1D–F). To determine whether trypsin addition had any side effects during PR8 infection, the expression of IL-6 and protease-activated receptors 2 (PAR2) was investigated. It was observed that trypsin had no significant effect on IL-6 mRNA or PAR2 protein level in response to PR8 infection (Additional file 1). These results indicated that PR8 infection can significantly induce the expression of NMBR in host cells.

### PR8 infection increases NMB and NMBR expression in vivo

To better understand the potential roles of NMB and NMBR in the host innate immune response to IAV infection, mice were infected with PR8, and lung tissues of PR8-infected and non-infected control animals were collected at 3 dpi. The tissue-specific expression of NMB and NMBR mRNA was assessed by RT-PCR and qRT-PCR. Similarly to above, it was observed that the expression of NMB and NMBR increased in lung tissues following infection with PR8 (Figures 1G–I).

### Cells deficient in NMBR are more susceptible to PR8 infection

**Figure 2:**
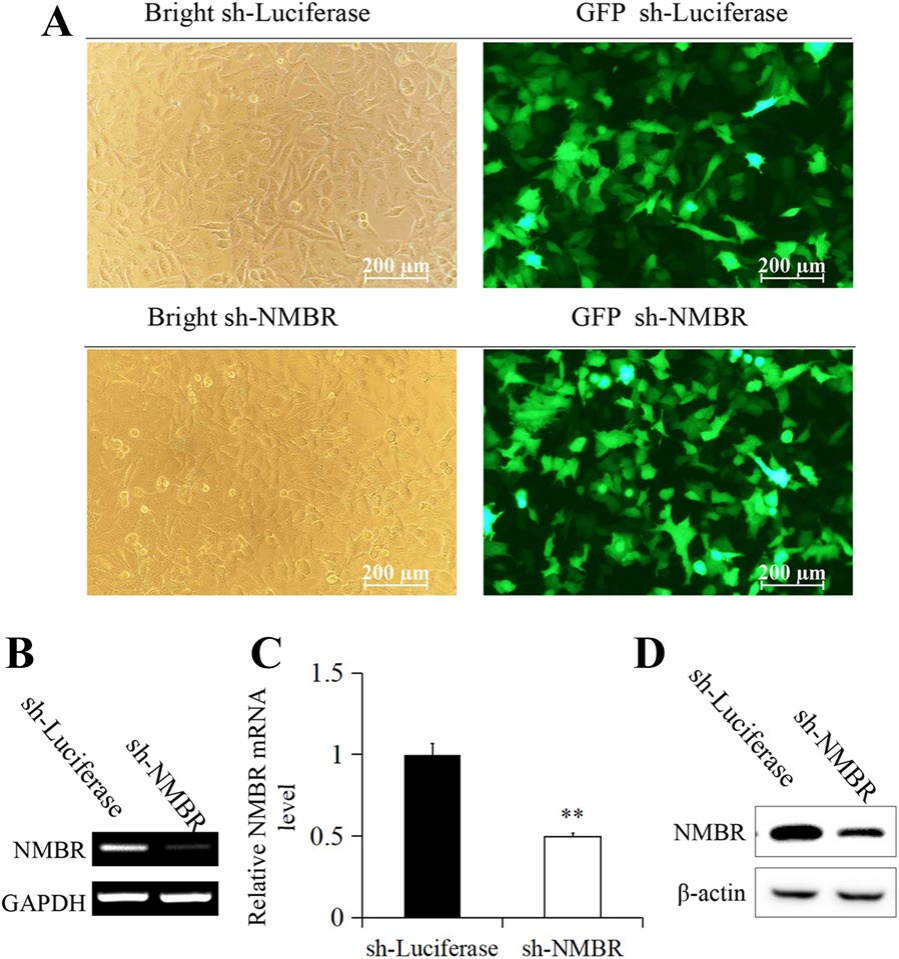
**Stable transfection of A549 cells with NMBR shRNA**. **A** Bright-field microscopy of cells transfected with shRNA-Luciferase (1, mock cells; 3, sh-NMBR cells); GFP-expressing cells were successfully transfected with shRNA-Luciferase (2, mock cells; 4, sh-NMBR cells). The expression of NMBR was assessed by RT-PCR (**B**), qRT-PCR (**C**) and WB (**D**). β-Actin or GAPDH was used for internal standardization. All images were captured at ×200 magnification, scale bars = 200 μm, **P < 0.05.

To test the hypothesis that NMB/NMBR plays a role in the host defences against IAV infection, A549 cells deficient in NMBR were generated. The transfection efficiency of the shRNA expression plasmid vector used in this study, as determined by the proportion of GFP-positive cells, is presented in Figure 2A. To verify the efficacy of the shRNA-mediated knockdown of NMBR in A549 cells, the expression of NMBR mRNA in sh-Luciferase controls and sh-NMBR cells was examined by RT-PCR and qRT-PCR (Figures 2B, C), and protein expression was examined by Western blotting (Figure 2D). The data demonstrated that shRNA targeting of NMBR efficiently silenced the expression of NMBR in the A549 cell line.

**Figure 3:**
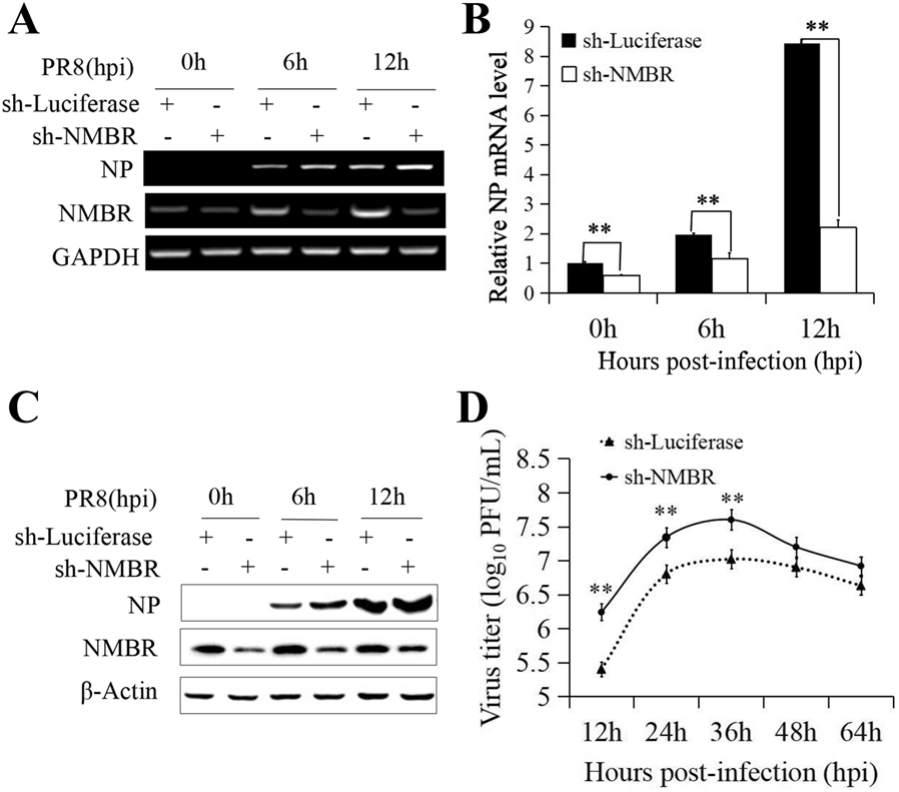
**Relative susceptibility of NMBR-deficient cells to PR8 infection.** The sh-NMBR cells and sh-Luciferase cells were infected with PR8 (MOI = 1) and collected at 0, 6, and 12 hpi. The expression of NP was assessed by RT-PCR (**A**), qRT-PCR (**B**) and WB (**C**). Culture supernatants were collected to test the titres by plaque assay at 12, 24, 36, 48 and 64 hpi (**D**). GAPDH was used as the reference housekeeping gene for internal standardization. **P < 0.01.

**Figure 4:**
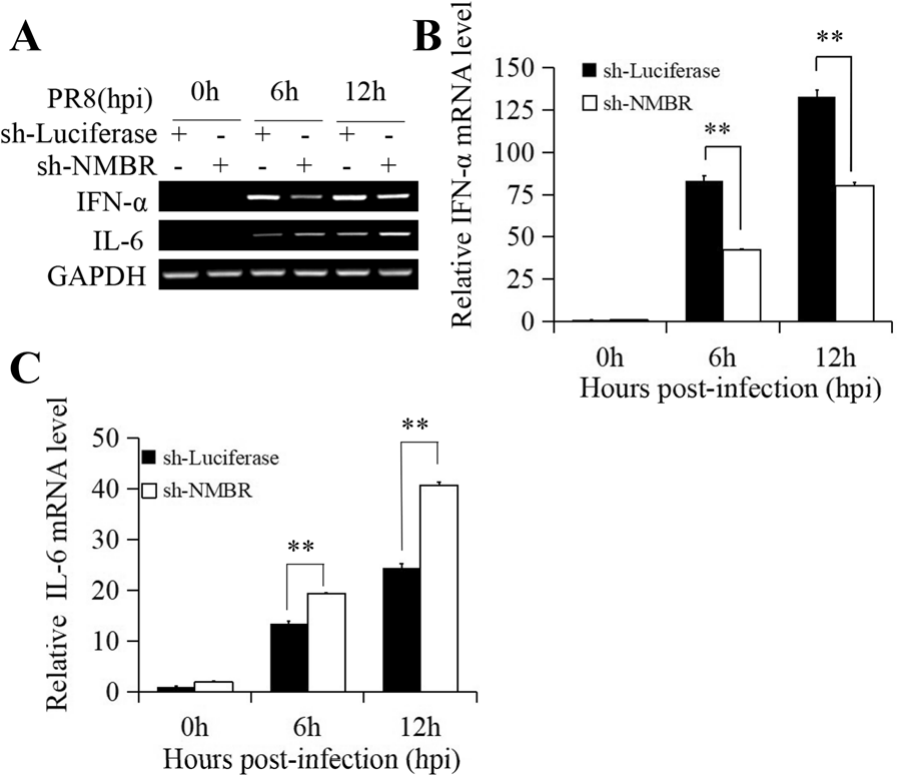
**Cytokine expression in cells lacking NMBR after PR8 infection.** The cells were infected with PR8 (MOI = 1) and harvested to assay the expression of IFN-α and IL-6 at 0, 6, and 12 hpi. **A** RT-PCR revealed a significant decrease in IFN-α expression and a significant increase in IL-6 expression in sh-NMBR cells compared to mock cells (sh-Luciferase cells); qRT-PCR confirmed the expression profile of **B** IFN-α and **C** IL-6. **P < 0.01, *P < 0.05.

Next, the sh-NMBR and sh-Luciferase control cells were infected with PR8 and harvested at 0, 6, and 12 hpi. NP expression was slightly enhanced in the NMBR-deficient cells compared to the mock-treated cells (Figure 3A). To confirm the reliability of these data, qRT-PCR was performed to measure the expression of NP, and a more significant result was obtained from this assay (Figure 3B). Similarly, the Western blot results showed that the expression of NP was substantially upregulated in the NMBR-deficient cells (Figure 3C). In addition, virus replication in NMBR-deficient cells was higher than that of control cells in the plaque assay (Figure 3D). To demonstrate the potential impact of NMBR signalling on the host innate immune system, the expression of IFN-α and IL-6 in the context of PR8 infection was assessed in sh-NMBR cells by RT-PCR. The data indicated a decrease in IFN-α expression, whereas the expression of IL-6 was upregulated in these cells (Figure 4A). The observed gene expression profile of IFN-α and IL-6 was further confirmed by qRT-PCR (Figures 4B, C). Taken together, these data suggest that NMBR is an important component in the host innate defence against IAV infection.

### NMB/NMBR inhibits PR8 infection in vitro

**Figure 5:**
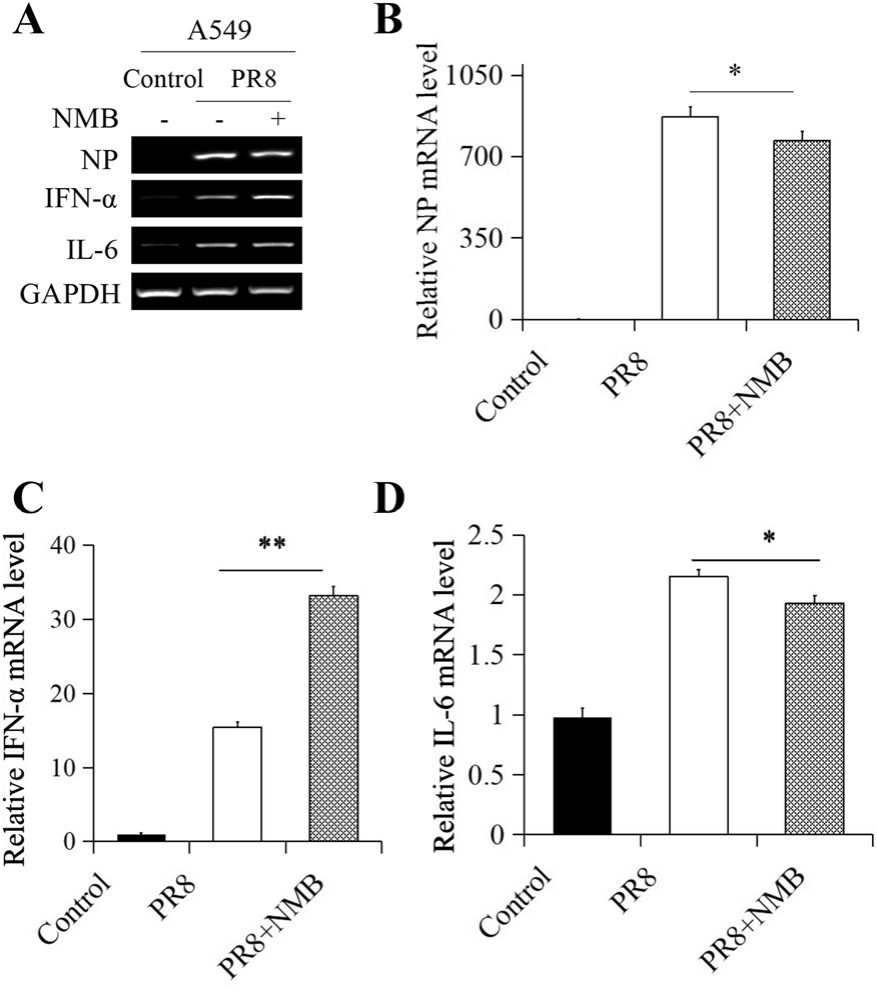
**Effect of NMB treatment on the expression of viral NP and cytokines in A549 cells.** The A549 cells were mock-treated or treated with NMB after infection with PR8 (MOI = 1). NMB-treated cells were harvested at 16 hpi. **A** The mRNA levels of NP, IFN-α, and IL-6 were measured by RT-PCR. qRT-PCR measurement of NP (**B**), IFN-α (**C**) and IL-6 (**D**) mRNA expression. GAPDH was used as the reference housekeeping gene for internal standardization. **P < 0.01, *P < 0.05.

**Figure 6:**
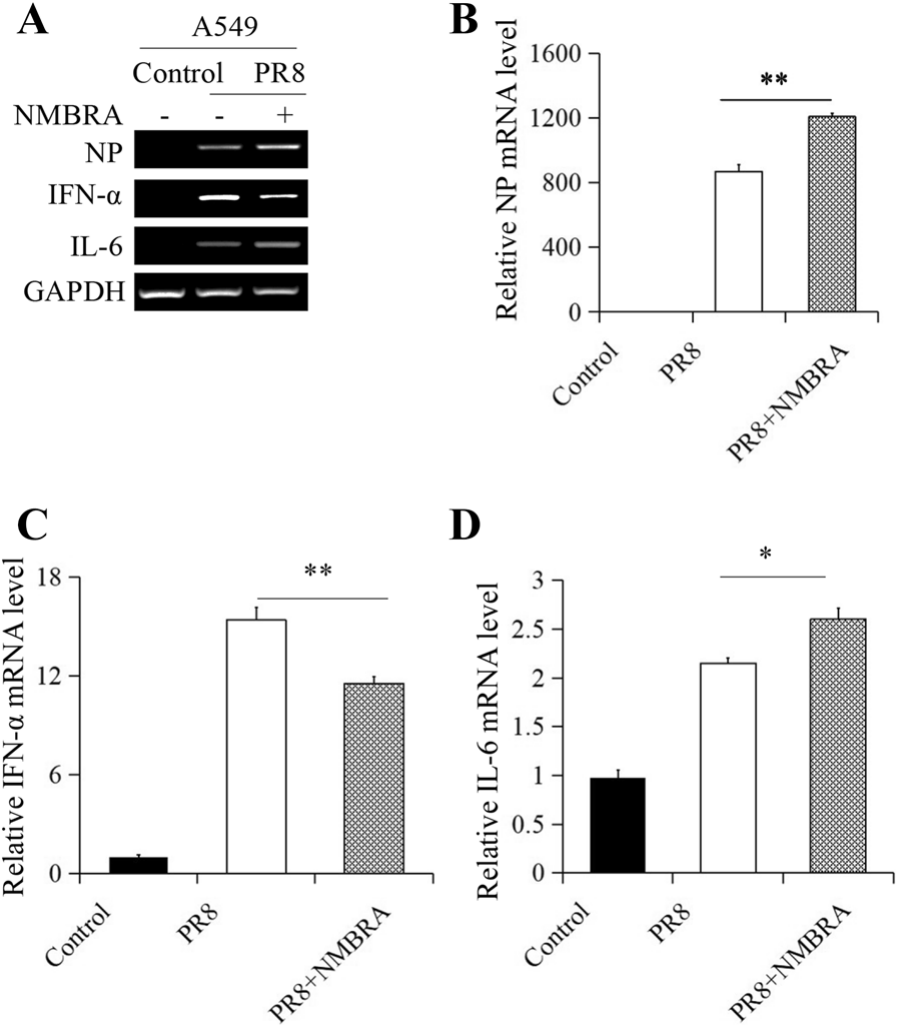
**Effect of NMBRA treatment on the expression of viral NP and cytokines in A549 cells.** The A549 cells were mock-treated or treated with NMBRA after infection with PR8 (MOI = 1). NMBRA-treated cells were harvested at 16 hpi. **A** The mRNA levels of NP, IFN-α, and IL-6 were measured by RT-PCR. The expression of NP (**B**), IFN-α (**C**), and IL-6 (**D**) were measured by qRT-PCR. GAPDH was used as the reference housekeeping gene for internal standardization. **P < 0.01, *P < 0.05.

To determine the effect of NMB on PR8 infection, the effects of NMB and NMBRA on the expression of viral NP, IFN-α and IL-6 were examined in A549 cells and BMDMs. NP expression was significantly reduced following NMB treatment in A549 cells (Figures 5A, B). Furthermore, treatment with NMB resulted in an upregulation of IFN-α (Figures 5A, C) and downregulation of IL-6 expression (Figures 5A, D) in PR8-infected A549 cells. Indeed, viral NP expression was increased in A549 cells treated with 1 nM NMBRA (Figures 6A, B). The expression of IFN-α in A549 cells was markedly downregulated (Figures 6A, C), and IL-6 was upregulated (Figures 6A, D) by NMBRA treatment after PR8 infection. In BMDMs, similar expression levels of NP, IFN-α and IL-6 mRNA induced by NMB treatment or NMBRA treatment were observed in BMDMs after PR8 infection (Additional files 2, 3). Thus, the expression profile of these genes in the presence of NMBRA was in contrast to that of NMB-treated cells. Meanwhile, the expression of IL-6 mRNA and PAR2 in BMDMs and the expression of NMBR in A549 cells and BMDMs were not affected by trypsin during PR8 infection (Additional file 4), suggesting that trypsin is not involved in the effects of NMB and NMBRA treatments in PR8-infected cells. Together, the results reveal that NMB/NMBR induced a substantial increase in IFN-α and a significant decrease in IL-6 expression in response to PR8 infection.

### NMB/NMBR exhibits anti-influenza virus activity in vivo

**Figure 7:**
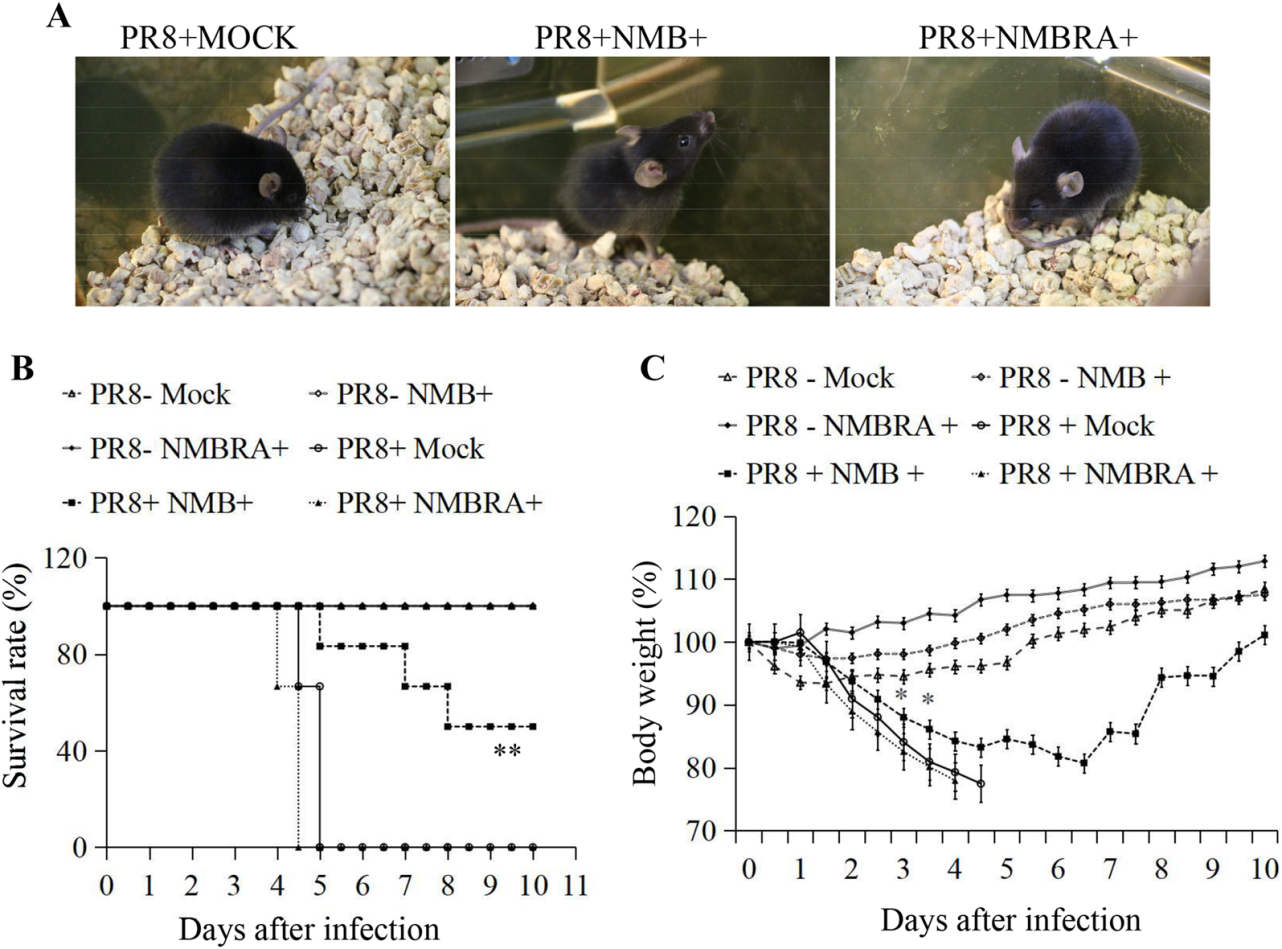
**Effects of NMB and NMBRA treatment on virus replication in vivo. A** Representative photographs of mock-treated mice infected with PR8, PR8-infected mice treated with 1 nM NMB, and PR8-infected mice treated with 1 nM NMBRA; **B** survival rates of mice in 6 treatment groups; **C** body weights of the 6 groups of mice. The mice were monitored for up to 10 days, and survival curves were compared using a log-rank test (GraphPad Prism 5). **P < 0.01, *P < 0.05.

**Figure 8:**
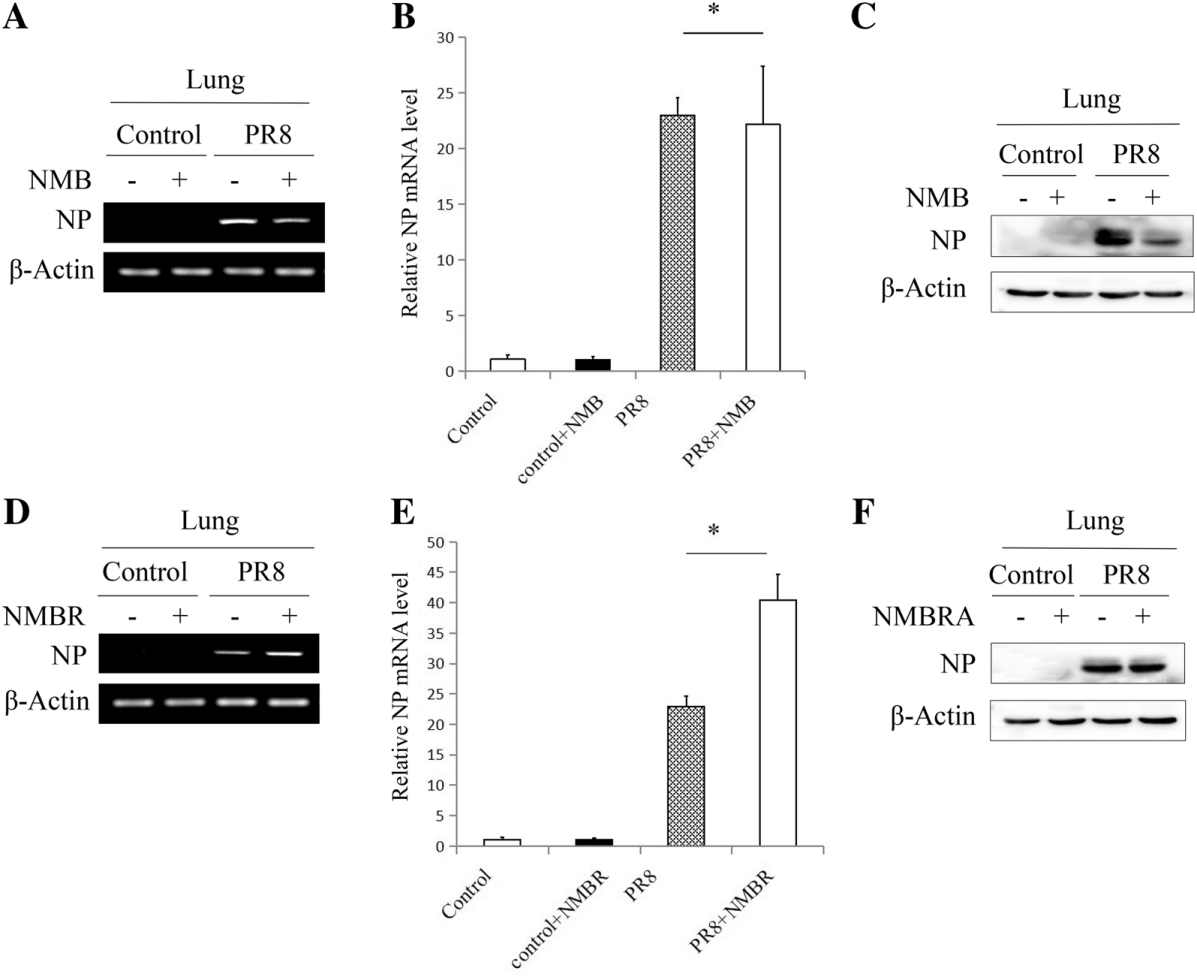
**Effects of NMB and NMBRA treatments on the expression of virus NP in mice.** Lung tissues from treated mice were sampled at 3 dpi. The profiles of NP expression were assayed by RT-PCR, qRT-PCR or Western blotting. Decreased virus NP expression after NMB treatment was observed (**A**–**C**); increased virus NP expression induced by NMBRA treatment was confirmed (**D**–**F**). β-Actin was used for internal standardization. *P < 0.05.

To investigate the anti-IAV activity of NMB/NMBR in vivo, mice were infected with PR8 and then injected with NMB, NMBRA, or mock control. As expected, PR8 challenge resulted in clinical signs consistent with PR8 infection in mice at 3 dpi (Figure 7A). However, the clinical signs were markedly less severe in animals treated with NMB and were exacerbated in the NMBRA-treated group (Figure 7A). To further determine the effects of NMB or NMBRA in vivo, mortality rates were compared between the groups over the 10-day period following PR8 challenge. As can be seen in Figure 7B, the control mice began to die by 4 dpi, and all mice succumbed by 5 dpi. However, mortality was reduced to 50% in the NMB-treated mice. Similar to the infection control group, all mice died in the NMBRA treatment group within 4.5 days after infection. Notably, all mice in the mock group survived the duration of the experiment. As presented in Figure 7C, the weight gain of all mice increased steadily in the three PR8-negative groups. Mice infected with PR8 exhibited a precipitous weight loss after 1 day, decreasing to less than 75% after 5 days. Mice infected with PR8 and treated with NMBRA lost weight faster than the PR8 infection-alone group. Interestingly, mice treated with NMB after virus challenge exhibited a steady weight gain after 7 days of continual weight loss. Consistent with the data from the above in vitro experiments, the lung tissues were sampled for analysis of viral NP expression, and NMB treatment resulted in a downregulation of NP expression following PR8 infection (Figures 8A, B). Analysis of protein expression by Western blotting confirmed that NMB treatment effectively inhibited the expression of NP protein in the lungs of mice infected with PR8 (Figure 8C). Moreover, the expression profiles of viral NP mRNA and protein after treatment with NMBRA were also assessed by RT-PCR, qRT-PCR and Western blotting. The expression of NP in the NMBRA-treated animals was opposite to that observed in the NMB-treated group (Figures 8D–F). Taken together, these data suggest that the expression of NMB could reduce the susceptibility of mice to PR8 infection.

**Figure 9:**
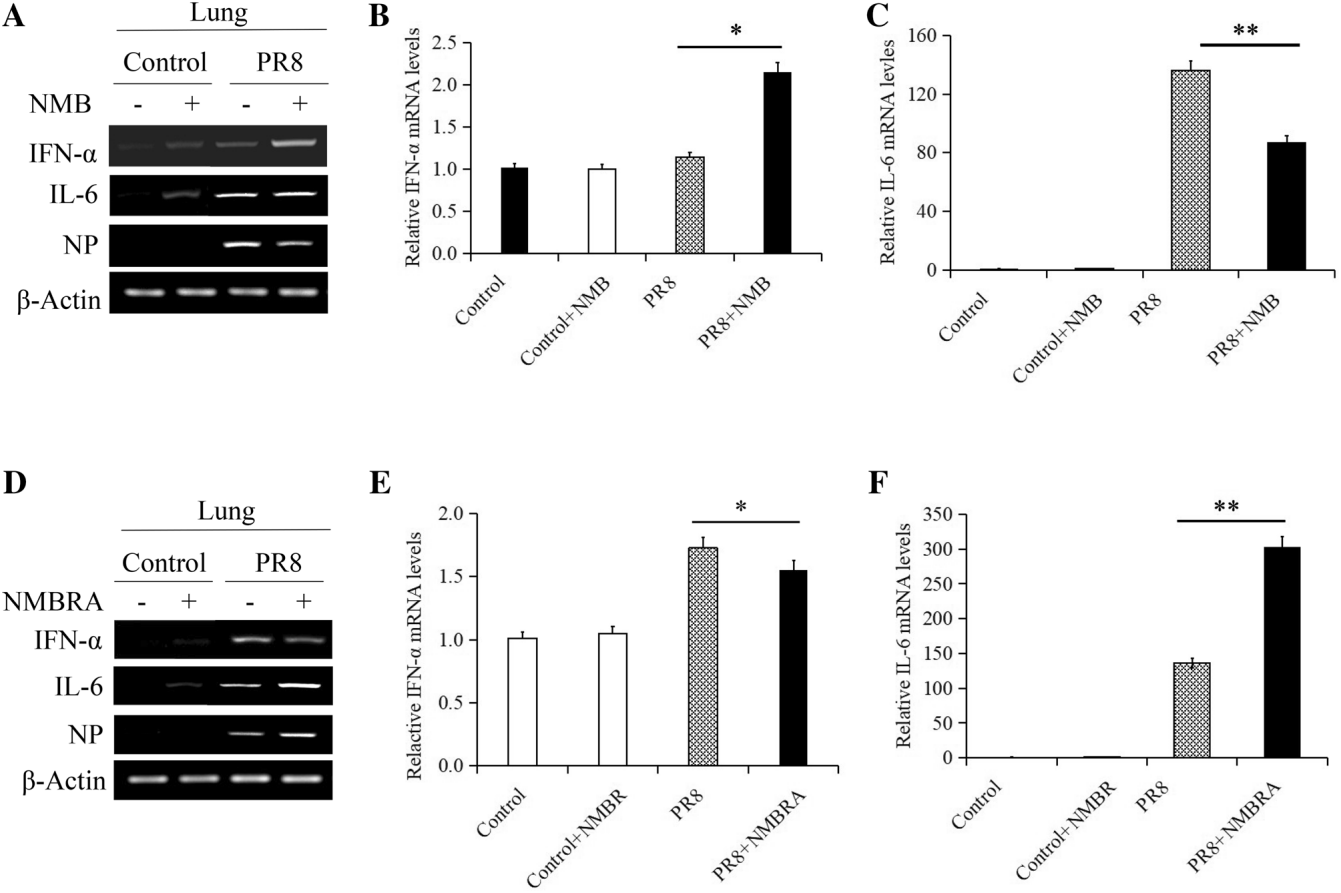
**Effects of NMB and NMBRA treatment on the expression of cytokines in mice.** Lung tissues from treated mice were sampled at 3 dpi. The expression patterns of IFN-α and IL-6 in lung tissues were assessed using RT-PCR and qRT-PCR. NMB treatment induced the upregulation of IFN-α (**A**, **B**) and downregulation of IL-6 (**A**, **C**); NMBRA treatment induced the downregulation of IFN-α (**D**, **E**) and upregulation of IL-6 (**D**, **F**). β-Actin was used as the reference housekeeping gene for internal standardization *P < 0.05; **P < 0.01.

Next, RT-PCR and qRT-PCR were used to determine the expression of IFN-α and IL-6 in mice infected with PR8 alone or in the context of NMB or NMBRA treatment. The combination of PR8 infection and NMB treatment induced a significant increase in IFN-α expression (Figures 9A, B) and decreased IL-6 expression (Figures 9A, C). Specifically, RT-PCR and qRT-PCR analysis indicated that NMBRA treatment induced a substantial decrease in IFN-α and a significant increase in IL-6 expression (Figures 9D–F). Thus, the effects of NMBRA were in contrast to those of NMB in PR8-infected mice, as expected.

## Discussion

A key member of the neuroendocrine system, NMB controls various exocrine and endocrine functions through signalling cascades upon binding to its receptor, NMBR [20, 28]. Although research into the antiviral activity of this protein in mammals has not been thoroughly examined, it has been well characterized in the context of immune regulation [19]. However, the expression profiles of NMB and NMBR during IAV infection and the activity of NMB/NMBR in hosts during virus infection remain unclear. The present study reports, for the first time, the expression and activity of NMB/NMBR in response to PR8 infection.

Interestingly, the upregulation of NMB and NMBR was detected after the virus challenge. A significant increase in NMBR expression was observed in the infected host. These data indicate that NMBR may be involved in the pathological or host defence processes of PR8/IAV infection. It is well known that many viruses are capable of modulating the expression of inflammatory cytokines (e.g., IL-6), thus allowing the virus to establish a successful infection [29–31]. Although it appears from the data presented here that induction of NMBR in response to IAV/PR8 infection is indeed part of the host innate response and is detrimental to the virus, further studies are necessary to confirm these findings.

Strikingly, the data presented here demonstrated that the disruption of NMBR signalling resulted in increased PR8 replication in vitro. This conclusion was supported by the fact that the depletion of NMBR increased the susceptibility of host cells to PR8 infection. Although the roles of IFN-α are beneficial for the host following viral infection, the excessive production of IL-6 stimulated by IAV infection can result in pathogenic effects in the host organs, increasing the risk of severe disease and death [32, 33]. Here, it was observed that the increased expression of IL-6 and decreased expression of IFN-α were induced in NMBR-deficient cells in response to PR8 infection. It is very likely that the high expression level of IL-6 and reduced IFN-α expression in cells lacking NMBR are contributing factors in the pathogenesis of PR8. Our results indicate that endogenous NMBR is an important part of the host defence against PR8 infection. Therefore, we hypothesized that the NMBR ligand, NMB, is a potential therapeutic agent for the treatment of PR8 infection.

To test the above hypothesis, the anti-influenza virus activity of NMB was examined both in vitro and in vivo. The data collected from all of the experiments that were conducted corroborated the hypothesis that NMB can inhibit IAV replication. As expected, NMB treatment reduced the clinical signs associated with PR8 infection. These results suggest that NMB is an important factor in combating PR8 infection. However, the specific antiviral mechanisms of NMB remain unclear. In the present study, NMB suppressed the expression of IL-6 in PR8-infected cells and mice. Since IL-6 plays a vital role in the pathogenesis of H1N1 [34], this may, at least in part, explain the anti-PR8 effect of NMB. It has been established that there is cross-talk between IL-6 signalling and IFN-α/β signalling [35–38]. The rapid induction of type I IFN is a critical step in the establishment of the innate antiviral response [17, 18]. Consistent with this concept, NMB treatment reversed the expression profiles of IFN-α and IL-6 induced by PR8 infection both in vitro and in vivo. Furthermore, when synthetic NMBRA was injected into mice after PR8 infection, the cytokine expression profiles reversed relative to those under NMB treatment, thus corroborating the observed antiviral activity of NMB. Therefore, it is likely that NMB plays an anti-inflammatory role via the regulation of the host type I IFN signalling pathway. Of course, the signalling pathway in which NMB participates requires further research.

The present data represent the first demonstration of the in vitro and in vivo efficacy of NMB/NMBR against PR8 infection. Cells deficient in NMBR expression exhibited increased susceptibility to the influenza A virus. Furthermore, the NMB/NMBR system appeared to increase IFN-α expression and decrease IL-6 expression after PR8 infection, which may serve to establish an antiviral state in the host. The effects observed following PR8 infection and NMBRA treatment further suggest an important role of NMB in antiviral immunity. However, the potential activity of NMB/NMBR against other viruses requires further research. These results provide a critical basis for the future therapeutic application of NMB/NMBR against IAV infection.

## Supplementary information

**Additional file 1. Effect of trypsin on the expression of IL-6 and PAR2 in cells.** The 293T and A549 cells were infected with PR8 (MOI = 1) in the presence or absence of trypsin. The 293T cells at 0, 3 and 12 hpi and A549 cells at 0, 3, 12, and 16 hpi were collected to test the expression of IL-6 mRNA in 293T and A549 cells by RT-PCR and qRT-PCR and the expression of PAR2 and NMBR in A549 cells by Western blotting. (A, B) IL-6 mRNA expression in 293T cells. (C) PAR2 expression in 293T cells. (D, E) IL-6 mRNA expression in A549 cells. (F) PAR2 expression in A549 cells. GAPDH and β-actin were used as the reference housekeeping genes for internal standardization.

**Additional file 2. Effect of NMB treatment on the expression of viral NP and cytokines in BMDMs.** The BMDMs were mock-treated or treated with NMB after infection with PR8 (MOI = 1). NMB-treated cells were harvested at 16 hpi. The mRNA levels of NP, IFN-α, and IL-6 were measured by RT-PCR (A). qRT-PCR measurement of NP (B), IFN-α (C) and IL-6 mRNA expression (D). β-Actin was used as the reference housekeeping gene for internal standardization. ** *P* < 0.01.

**Additional file 3. Effect of NMBRA treatment on the expression of viral NP and cytokines in BMDMs.** The BMDMs were mock-treated or treated with NMBRA after infection with PR8 (MOI = 1). NMBRA-treated cells were harvested at 16 hpi. mRNA levels of NP, IFN-α, and IL-6 measured by RT-PCR (A). The expression of NP (B), IFN-α (C), and IL-6 (D) was measured by qRT-PCR. β-Actin was used as the reference housekeeping gene for internal standardization. **P < 0.01.

**Additional file 4. Effect of trypsin on the expression of IL-6, PAR2 and NMBR in vitro.** The BMDMs and A549 cells were infected with PR8 (MOI = 1) in the presence or absence of trypsin. The BMDMs and A549 cells were collected at the indicated times to test the expression of IL-6 mRNA by RT-PCR and qRT-PCR and the expression of PAR2 and NMBR by Western blotting. (A, B) IL-6 mRNA expression in BMDMs. (C) PAR2 and NMBR expression in BMDMs. (D) NMBR expression in A549 cells. β-Actin was used as the reference housekeeping gene for internal standardization.

## Abbreviations

BMDMs: bone marrow-derived macrophages
dpi: days post-infection
hpi: hours post-infection
IAV: influenza A virus
IFN-α/β: type I interferon
MOI: multiplicity of infection
NMB: neuromedin B
NMBR: neuromedin B receptor
NMBRA: NMBR antagonist
NP: nucleoprotein
PAR2: protease-activated receptors 2
PFU: plaque-forming unit
qRT-PCR: quantitative real-time RT-PCR
shRNA: short hairpin RNA
SPF: specific pathogen-free
