# Supplementary figures and images for: Role of neuromedin B and its receptor in the innate immune responses against influenza A virus infection in vitro and in vivo
Source: Vet Res. 2019 Oct 10;50:80. doi: 10.1186/s13567-019-0695-2 (PMC6785861; doi:10.1186/s13567-019-0695-2)

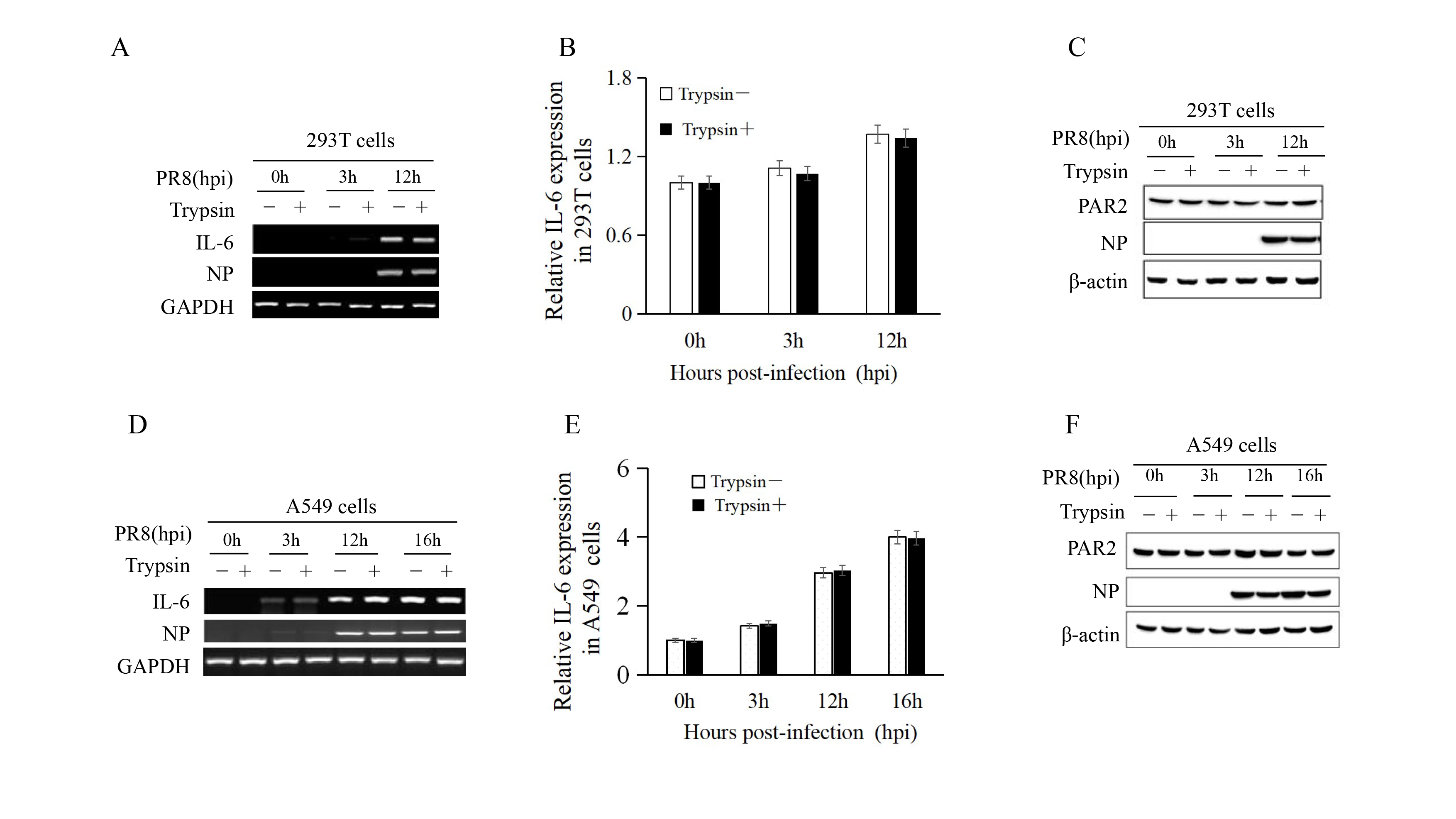

Supplement: Supplementary file 1 — Additional file 1. Effect of trypsin on the expression of IL-6 and PAR2 in cells. The 293T and A549 cells were infected with PR8 (MOI = 1) in the presence or absence of trypsin. The 293T cells at 0, 3 and 12 hpi and A549 cells at 0, 3, 12, and 16 hpi were collected to test the expression of IL-6 mRNA in 293T and A549 cells by RT-PCR and qRT-PCR and the expression of PAR2 and NMBR in A549 cells by Western blotting. (A, B) IL-6 mRNA expression in 293T cells. (C) PAR2 expression in 293T cells. (D, E) IL-6 mRNA expression in A549 cells. (F) PAR2 expression in A549 cells. GAPDH and β-actin were used as the reference housekeeping genes for internal standardization. [file 13567_2019_695_MOESM1_ESM.tif]

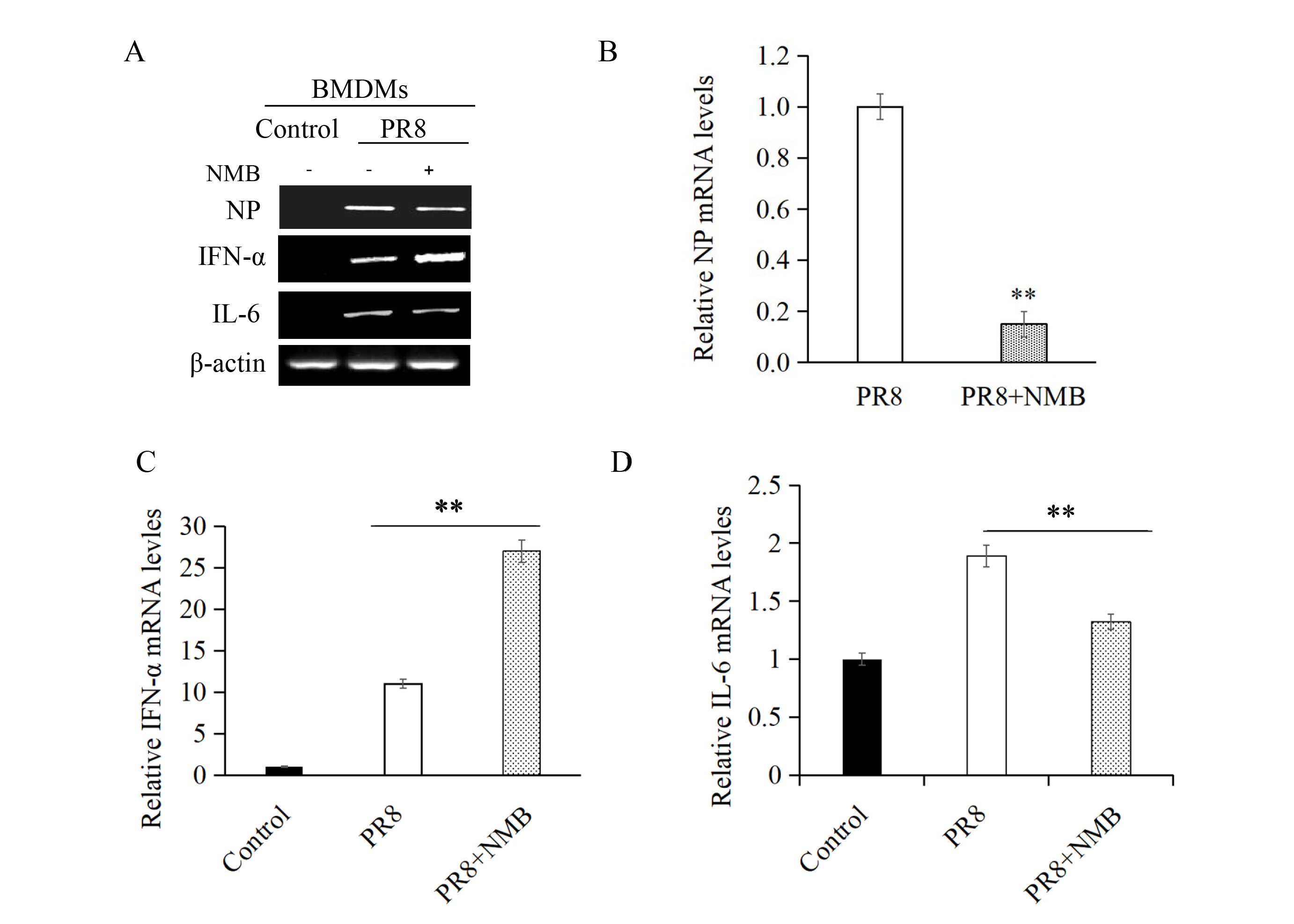

Supplement: Supplementary file 2 — Additional file 2. Effect of NMB treatment on the expression of viral NP and cytokines in BMDMs. The BMDMs were mock-treated or treated with NMB after infection with PR8 (MOI = 1). NMB-treated cells were harvested at 16 hpi. The mRNA levels of NP, IFN-α, and IL-6 were measured by RT-PCR (A). qRT-PCR measurement of NP (B), IFN-α (C) and IL-6 mRNA expression (D). β-Actin was used as the reference housekeeping gene for internal standardization. ** P < 0.01. [file 13567_2019_695_MOESM2_ESM.tif]

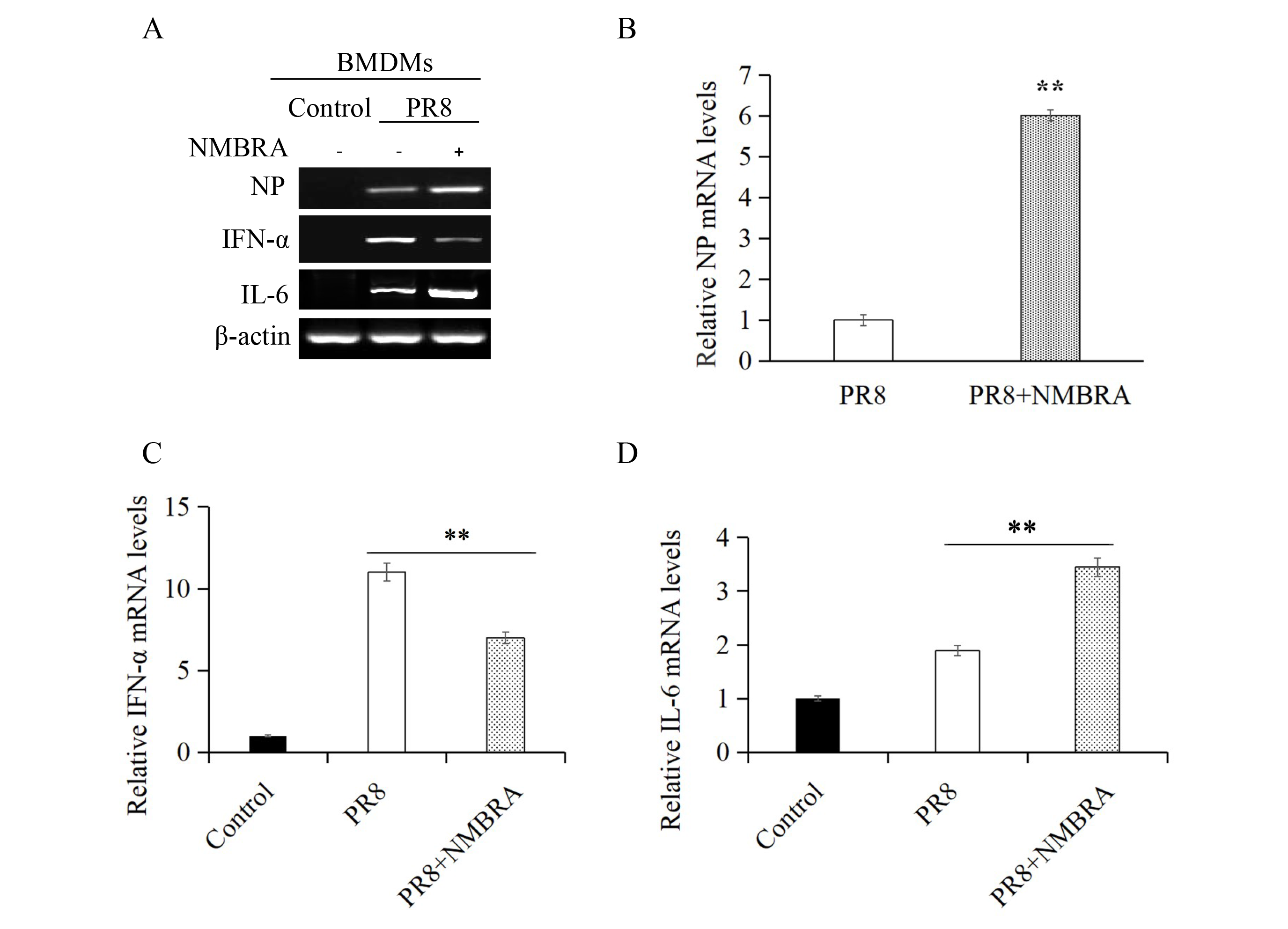

Supplement: Supplementary file 3 — Additional file 3. Effect of NMBRA treatment on the expression of viral NP and cytokines in BMDMs. The BMDMs were mock-treated or treated with NMBRA after infection with PR8 (MOI = 1). NMBRA-treated cells were harvested at 16 hpi. mRNA levels of NP, IFN-α, and IL-6 measured by RT-PCR (A). The expression of NP (B), IFN-α (C), and IL-6 (D) was measured by qRT-PCR. β-Actin was used as the reference housekeeping gene for internal standardization. **P < 0.01. [file 13567_2019_695_MOESM3_ESM.tif]

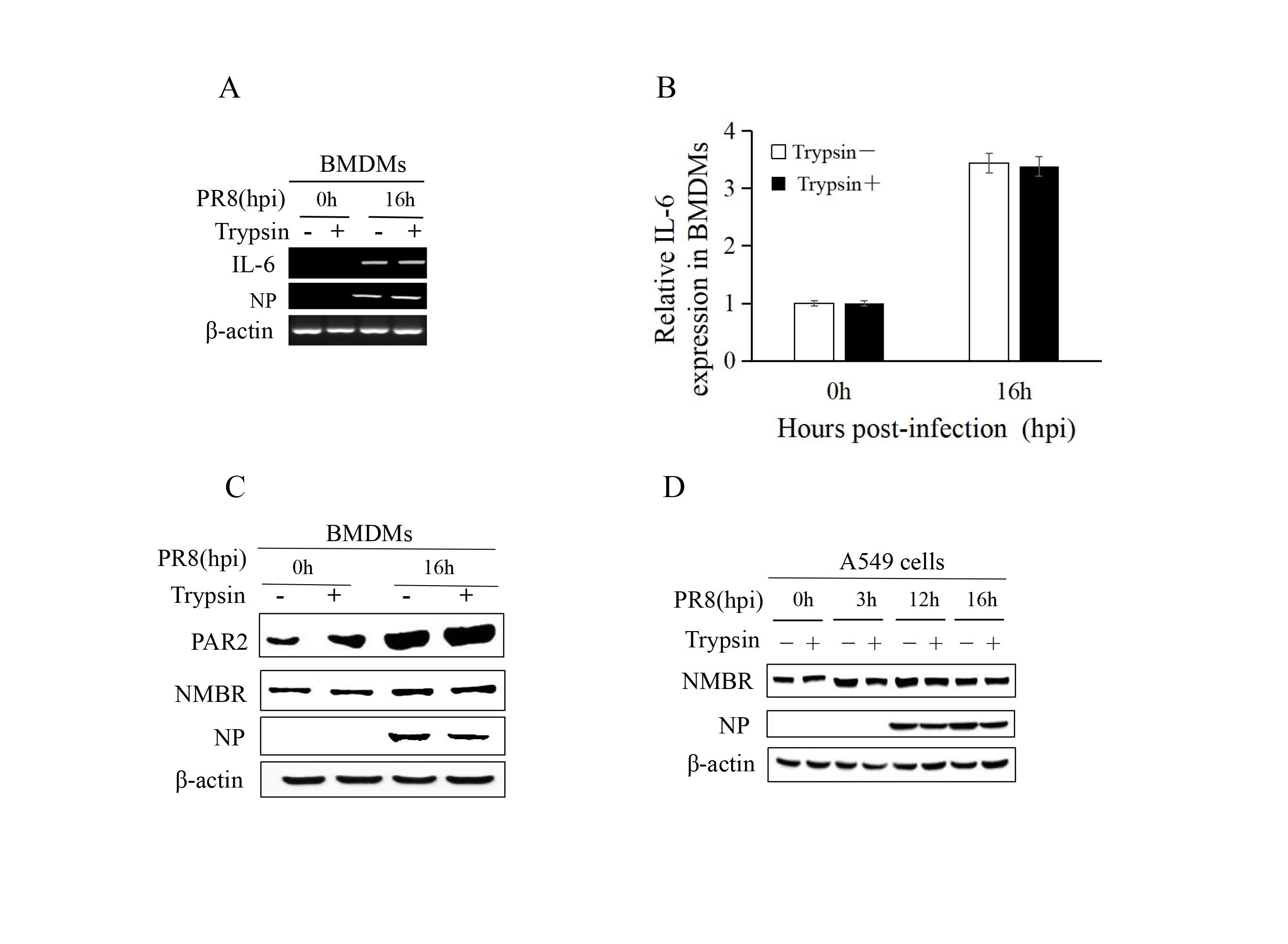

Supplement: Supplementary file 4 — Additional file 4. Effect of trypsin on the expression of IL-6, PAR2 and NMBR in vitro. The BMDMs and A549 cells were infected with PR8 (MOI = 1) in the presence or absence of trypsin. The BMDMs and A549 cells were collected at the indicated times to test the expression of IL-6 mRNA by RT-PCR and qRT-PCR and the expression of PAR2 and NMBR by Western blotting. (A, B) IL-6 mRNA expression in BMDMs. (C) PAR2 and NMBR expression in BMDMs. (D) NMBR expression in A549 cells. β-Actin was used as the reference housekeeping gene for internal standardization. [file 13567_2019_695_MOESM4_ESM.tif]
